# Seamless bathymetry and topography datasets for New South Wales, Australia

**DOI:** 10.1038/sdata.2018.115

**Published:** 2018-06-19

**Authors:** Kaya M. Wilson, Hannah E. Power

**Affiliations:** 1 School of Environmental and Life Sciences, University of Newcastle, Newcastle, Australia

**Keywords:** Environmental chemistry, Natural hazards, Geography

## Abstract

This paper describes three datasets of seamless bathymetry and coastal topography for Sydney Harbour (Port Jackson), Botany and Bate Bays, and the Hawkesbury River. The datasets used to form these compilations were the most recent and highest quality available to the authors and were originally collated using the software ESRI ArcGIS. The original compilation of this data was undertaken to support tsunami modelling research by the authors of this paper. Before processing, all data were adjusted and/or reprojected to conform to the vertical datum Australian Height Datum (AHD) and horizontal projection WGS84 UTM zone 56. Data resolution and density was highly variable and grid resolutions of the final datasets were selected as the highest resolutions possible using the most sparse data in the compilation in question. For areas where no data were available, the ESRI ArcGIS interpolation tool, *Topo to Raster*, was used to provide a best estimate. These dastasets of three important Australian waterways provide a useful tool for coastal research and scientific interest.

## Background & Summary

Seamless elevation datasets comprising nearshore bathymetry and coastal elevation are extremely useful for coastal process research, including morphological studies^1^ and coastal modelling^2^. Producing quality datasets requires some hydrographic expertise and, typically, licensed software to align multiple datasets from numerous sources into consistent datums and projections. Data quality and density may also be highly variable and there may be a lack of metadata. The datasets published as a compilation in this study have been collated from multiple sources and thoroughly assessed for data accuracy and quality. The authors have made these datasets publicly available to avoid replication of effort and to further research efforts in the region.

Geoscience Australia (GA) is the official Australian national repository for bathymetric data, however, the collection is not comprehensive. Data for the regions examined in this study are also held by several local and state authorities, including the New South Wales (NSW) Office of Environment and Heritage (OEH), the Port Authority of NSW, and NSW Roads and Maritime Services (RMS). The majority of data are held under creative commons licensing (CC BY) and are usually obtained by contacting the relevant authority with a request.

The standard method of handling Digital Elevation Models (DEMs) by Geographic Information Systems (GIS) and modelling software suites is to use a single resolution for the whole DEM as opposed to a variable resolution. This means that the most appropriate resolution selected for a single DEM is that of the more sparse datasets being compiled. Some data can be interpolated, however, interpolation reduces accuracy and should only be used if there is no measured data available.

If interpolation is necessary across areas with no data coverage, various techniques can be used. These techniques tend to take into account the surrounding datasets and suggest an estimate of missing data using different mathematical interpolations. The *Topo to Raster* tool in ESRI ArcGIS provides an efficient method of interpolation that is specifically designed for the creation of hydrologically correct DEMs. It is based on the ANUDEM program developed by Hutchinson^3,4^ and has been used for several continent wide DEM productions including Australia’s national 250 m resolution elevation grid^5^. *Topo to Raster* can be described as a discretised thin plate spline technique^6^ with adaptations made to the roughness penalty in order to provide continuity of terrain.

DEMs compiled from multiple sources can be updated with new datasets sampled to the same resolution, however, DEMs do not have the flexibility of raw data as some information is lost when the data is transposed, resampled, and/or compiled. Date of compilation and/or publication is therefore always relevant and should be referenced alongside DEM usage.

Data coverage was deemed to be sufficiently extensive to warrant the creation of a DEM for the geographical areas in this study but areas of interpolation must be viewed as estimation only and treated with the knowledge that major features may not be visible. In addition, the DEMs created in this study are for use in research and are not held to the standards and specifications of nautical charts published by hydrographic authorities. These DEMs are therefore not suitable to be used for navigational purposes.

**Figure 1:**
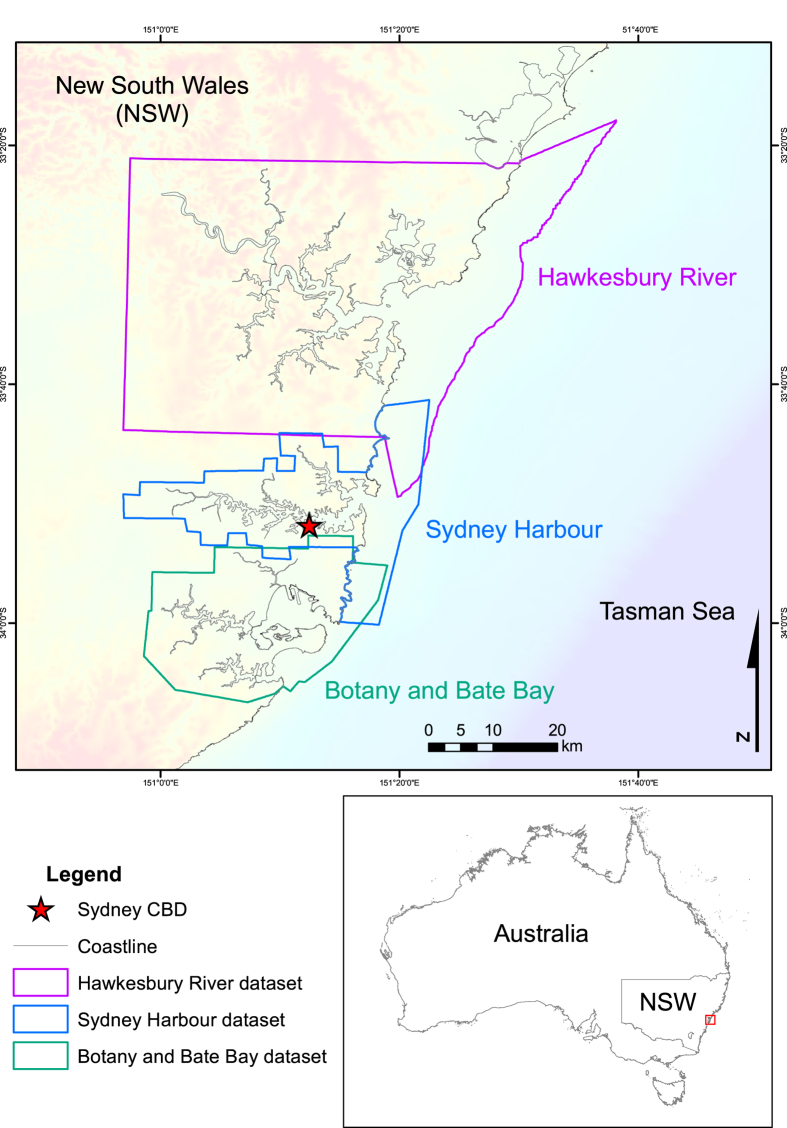
Dataset location map. Image created by KMW using ESRI ArcMap 10.3.1 (http://www.esri.com/arcgis/about-arcgis) with background elevation^5^ and coastline data^8^ from © Commonwealth of Australia (Geoscience Australia).

**Figure 2:**
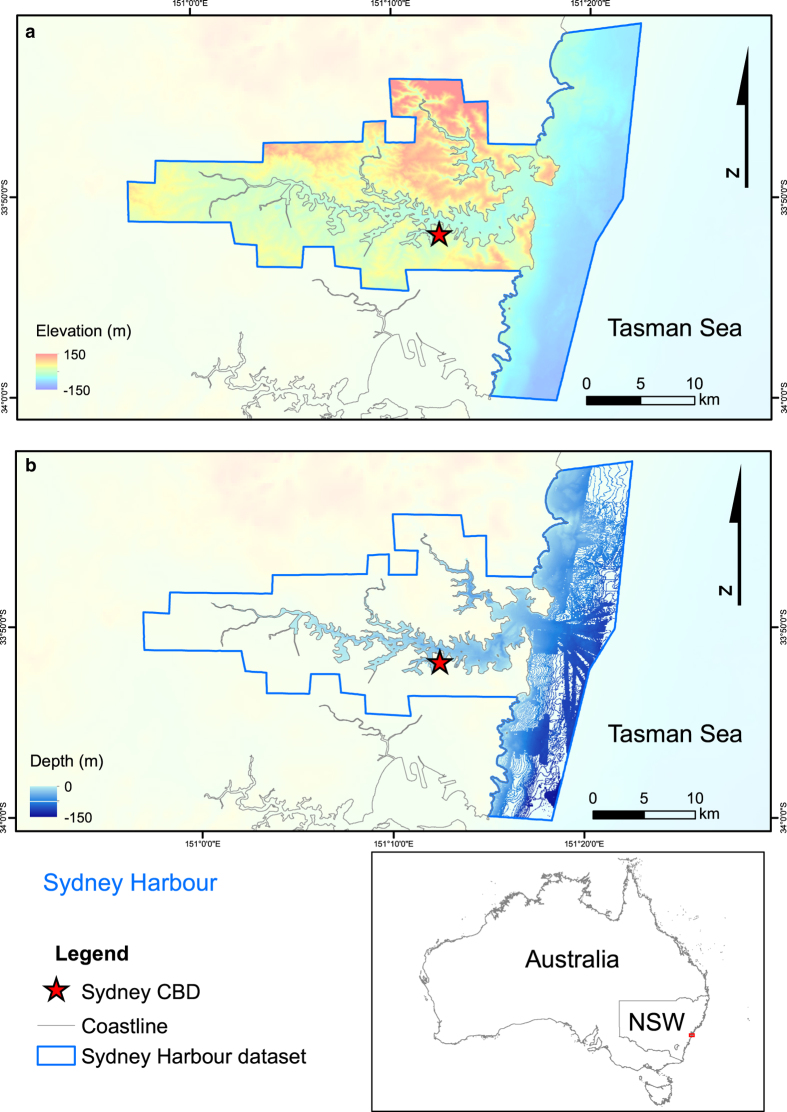
Map of Sydney Harbour (Port Jackson) compilation bathymetric and topographic dataset and the extent of the bathymetry data coverage. (**a**) Bathymetric and topographic dataset for Sydney Harbour. (**b**) Bathymetric data coverage for Sydney Harbour dataset. Image created by KMW using ESRI ArcMap 10.3.1 (http://www.esri.com/arcgis/about-arcgis) with elevation^5^ and coastline data^8^ from © Commonwealth of Australia (Geoscience Australia).

**Figure 3:**
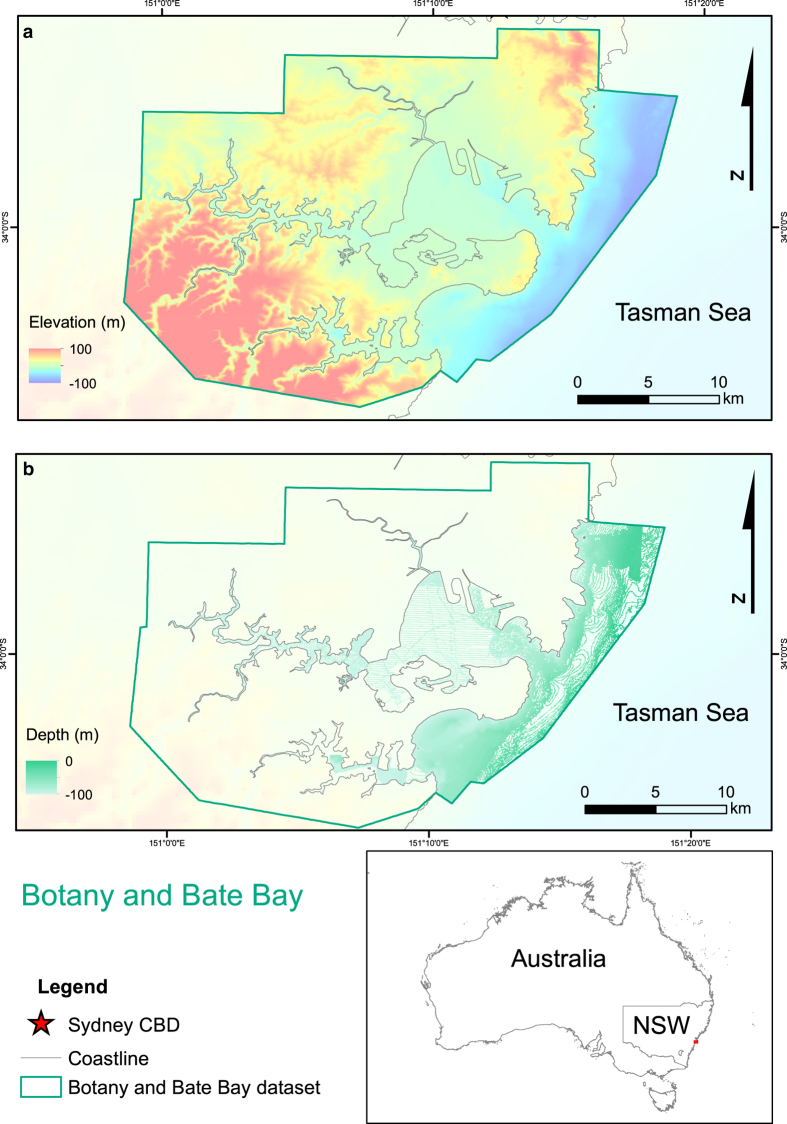
Map of Botany and Bate Bay compilation bathymetric and topographic dataset and the extent of the bathymetry data coverage. (**a**) Bathymetric and topographic dataset for Botany and Bate Bay. (**b**) Bathymetric data coverage for Botany and Bate Bay dataset. Image created by KMW using ESRI ArcMap 10.3.1 (http://www.esri.com/arcgis/about-arcgis) with elevation^5^ and coastline data^8^ from © Commonwealth of Australia (Geoscience Australia). Elevation data provided are relative to AHD.

**Figure 4:**
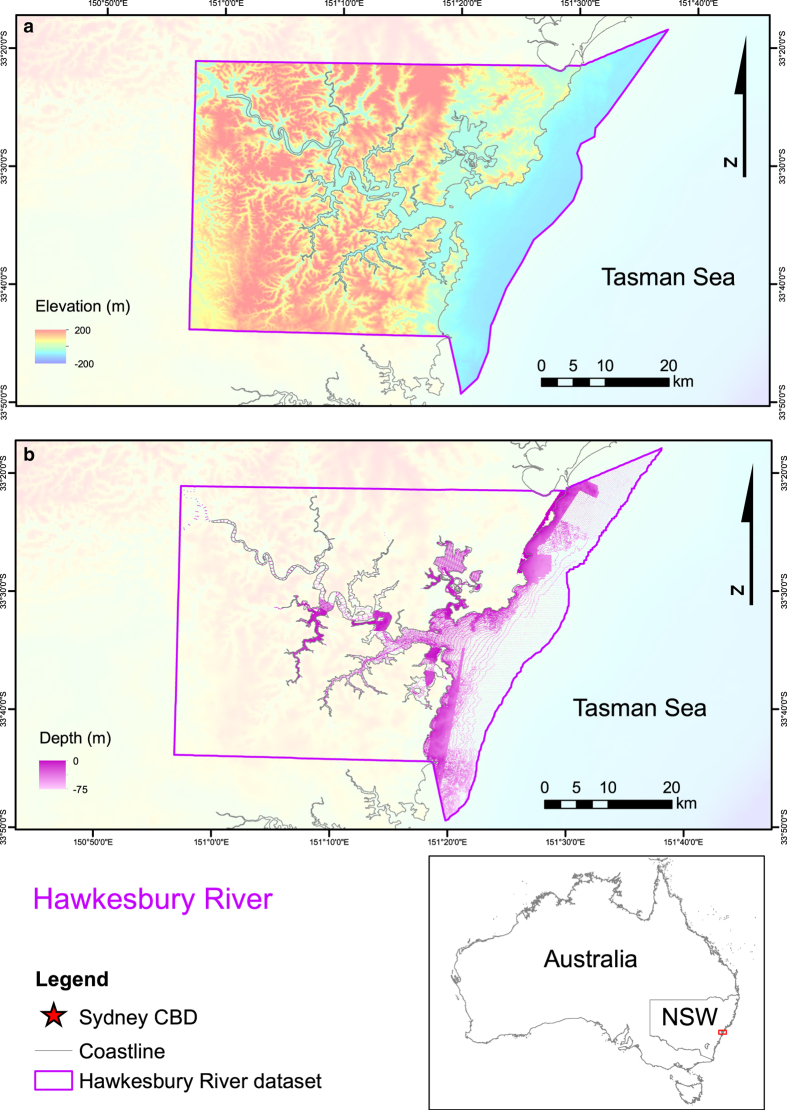
Map of Hawkesbury River compilation bathymetric and topographic dataset and the extent of the bathymetry data coverage. (**a**) Bathymetric and topographic dataset for the Hawkesbury River. (**b**) Bathymetric data coverage for the Hawkesbury River dataset. Image created by KMW using ESRI ArcMap 10.3.1 (http://www.esri.com/arcgis/about-arcgis) with elevation^5^ and coastline data^8^ from © Commonwealth of Australia (Geoscience Australia). Elevation data provided are relative to AHD.

In this paper, we present three study sites selected for the compilation of seamless, gridded elevation datasets originally compiled to support a larger tsunami modelling project^2^. The study sites selected were Sydney Harbour, Botany and Bate Bay, and the Hawkesbury River (Fig. 1). These major Australian waterways were selected based on their proximity to low lying development and bathymetric data availability. Topographic data were available in high resolutions for all locations. The seamless datasets are presented in Figs. 2, 3, 4. These datasets provide an additional tool for further research in these areas and aim to reduce replication of work and effort in the production of DEMs.

## Methods

The data used to compile the seamless elevation datasets in this paper were the most recent and highest quality available to the authors in 2016.

### Data Acquisition

Data were obtained from multiple sources and methods of acquisition (see Tables 1, 2, 3). In general, data were provided as a series of individual surveys. These surveys were derived from variable sources: singlebeam, multibeam, bathymetric lidar, and topographic lidar. Topographic datasets were collected to standards consistent with Australian Intergovernmental Committee on Surveying and Mapping (ICSM) LiDAR Acquisition Specifications and covered the entirety of each location, so did not require compilation or interpolation. Where bathymetric surveys overlapped, multibeam data and lidar data were preferenced over singlebeam data, which were often very sparse. If an overlapping dataset appeared anomalous and showed significant deviation from all other datasets, it was not included in the compilation. In general, metadata specifying the make and model of instrumentation used in data collection was not provided. Similarly, survey specifications were generally not provided. Therefore, we infer that the survey data and subsequent compilations do not meet minimum standards of any International Hydrographic Organisation (IHO) Order specifications for a hydrographic survey^7^.

### Data Processing

Bathymetry data were imported into the software ESRI ArcGIS 10.1™. The majority of data obtained was in point form and imported as x,y,z files. The vertical datum and horizontal projection were provided with the metadata and were assigned to the ESRI ArcGIS 10.1™ working files. The optimum gridded resolution for each individual dataset was then determined based on the density of the data. The point files were then converted to raster format and gridded at the chosen resolution.

Bathymetry raster datasets were converted to AHD vertical datum with depths below AHD described with a negative number and heights above AHD described with a positive number. Rasters were also projected using WGS84 UTM56S. Once all raster datasets for each area had been obtained, the rasters were mosaiced together using the *Mosaic to new Raster* tool. Rasters were prioritised based on data acquisition technique and the grid size chosen for the new raster was the largest grid size amongst the component rasters; therefore, the final grid size was the largest of all the component raster grid sizes.

Topography datasets were downloaded from the Geoscience Australia National Elevation Data Framework (http://www.ga.gov.au/elvis/). These datasets were reprojected into WGS84 UTM56S. The coastline dataset shapefile was also downloaded from Geoscience Australia^8^ and reprojected into WGS84 UTM56S.

Both the bathymetry and topography rasters were converted to point files. These point files were then input into the *Topo to Raster* tool as ‘Point Elevation’ data. The coastline shapefile was defined as a contour of elevation at 0 m AHD. Since *Topo to Raster* assumes a rectagonal coverage, the output raster was then clipped to coincide with the shape of the original data.

Datasets were also converted into WGS84 Geographic Coordinate System (GCS) to facilitate wider use and both GCS and UTM projected datasets were submitted to GeoMapApp (http://www.geomapapp.org/) and GMRT (https://www.gmrt.org/) databases. Users wishing to use this data on a more regional scale are recommended to explore these databases.

### Data Outputs

All three datasets are available in ESRI ASCII Raster format in both WGS84 GCS and projected in WGS84 UTM56S. Data points are provided to a precision of ≤0.001 m. Note that this is not a reflection of data accuracy.

Due to imperfect interpolation, some evidence of the original bathymetric survey data points may be visible in the datasets.

### Code availability

The version of ESRI used to create these datasets was ESRI ArcGIS 10.1™. The datasets were exported using newer versions of the software up to ESRI ArcGIS 10.5™. Using the same tools and methods described in this paper, identical datasets would be attainable using different versions of this software.

## Data Records

The dataset for Sydney Harbour is available in ESRI ASCII Raster format in both WGS84 GCS and projected in WGS84 UTM56S for download (Data Citation 1).

The dataset for Botany and Bate Bay is available in ESRI ASCII Raster format in both WGS84 GCS and projected in WGS84 UTM56S for download (Data Citation 2).

The dataset for the Hawkesbury River is available in ESRI ASCII Raster format in both WGS84 GCS and projected in WGS84 UTM56S for download at (Data Citation 3).

## Technical Validation

The topographic data used in these datasets were captured to standards that are generally consistent with the Australian ICSM LiDAR Acquisition Specifications, which require a fundamental vertical accuracy of at least 0.3 m (95% confidence) and horizontal accuracy of at least 0.8 m (95% confidence) as specified in the topography metadata (http://www.ga.gov.au/elvis). No additional validation was considered necessary.

Numerous bathymetric surveys collected with different specifications contributed to this dataset and the details of those specifications were generally not provided. Therefore, the standard methods of validating bathymetric data, such as calculation of Total Propagated Uncertainty (TPU) or considering IHO Order specifications, were not possible. The data were also gridded which, in places, reduces the resolution of the data. Locations where data overlapped provided some validation. Depth differences between datasets in shallower waters (<30 m) were within 1–2 m. Fewer datasets overlapped in deeper waters and depth differences in these regions were typically greater (1–5 m). Given the inability to assess the bathymetric data using standard methods of validation, the bathymetric data in these datasets should be considered unsuitable for navigation.

The proportion of the dataset that is comprised of original data and that which is interpolated should be taken into consideration when using these datasets. Original data was available for the full extent of the topographic regions of the dataset, meaning that data points were available for every grid cell in the dataset. The bathymetric datasets, however, varied in their degrees of coverage. Within the bathymetric domain of the datasets provided, for each grid cell the Sydney Harbour dataset had at least one data point in 77% of grid cells, Botany and Bate Bay, 70%, and the Hawkesbury River, 40%. The distribution of this data coverage can be viewed in Figs. 2b, 3b and 4b for each site respectively.

## Additional information

**How to cite this article**: Wilson, K. M. & Power, H. E. Seamless bathymetry and topography datasets for New South Wales, Australia. *Sci. Data* 5:180115 doi: 10.1038/sdata.2018.115 (2018).

**Publisher’s note**: Springer Nature remains neutral with regard to jurisdictional claims in published maps and institutional affiliations.

## Supplementary Material



## Figures and Tables

**Table 1:** Sydney Harbour compilation datasets.

Dataset	Data Provider	Data Acquisition Method	Date of Acquisition (if known)
5 m Digital Elevation Model (Sydney)	Geoscience Australia (GA), National Elevation Data Frame-work (NEDF)	Topographic lidar	2001–2015
CentralNSW_0_50_4	GA	Compilation	Up to 2012
CentralNSW_50_10000_1	GA	Compilation	Up to 2012
coo02by_v1	OEH	Multibeam	2002
syd5m	OEH	Multibeam	Before 2015
snb_2mby	OEH	Multibeam	Before 2015
snbs_2mby	OEH	Multibeam	Before 2015
BilgolaBeach2014Apr	OEH	Singlebeam	April, 2014
BilgolaBeach2014May	OEH	Singlebeam	May, 2014
BilgolaBeachAug	OEH	Singlebeam	August, 2014
BonginBay2014Apr	OEH	Singlebeam	April, 2014
BonginBay2014Aug	OEH	Singlebeam	August, 2014
BonginBay2014Feb	OEH	Singlebeam	February, 2014
DeeWhyLagoon2012	OEH	Singlebeam	2012
DeeWhyBeach2014Apr	OEH	Singlebeam	April, 2014
DeeWhyBeach2014Aug	OEH	Singlebeam	August, 2014
DeeWhyBeach2014Feb	OEH	Singlebeam	February, 2014
DeeWhyBeach2014May	OEH	Singlebeam	May, 2014
Manly2012	OEH	Singlebeam	2012
Narrabeen2011_jetski	OEH	Singlebeam	2011
Narrabeen2011_quad	OEH	Singlebeam	2011
Narrabeen2011_seascan	OEH	Singlebeam	2011
Sydney Harbour	RMS	Multiple	Up to 2015
All datasets were compiled or resampled to a grid size of 10 m.			

**Table 2:** Botany and Bate Bay compilation datasets.

Dataset	Data Provider	Data Acquisition Method	Date of Acquisition (if known)
1 second SRTM Derived Smoothed Digital Elevation Model (DEM-S) version 1.0	GA, NEDF	Satellite derived topography	2001–2015
jibb_littl	OEH	Multibeam	Before 2015
phent_by	OEH	Multibeam	Before 2015
ph_bb13	OEH	Multibeam	2013
merries_by	OEH	Multibeam	Before 2015
batebay75_by	OEH	Multibeam	Before 2015
Botany_20m	RMS	Multibeam	Up to 2015
BateBay_2011	OEH	Singlebeam	2011
CronullaOffshore2012	OEH	Singlebeam	2012
PortHacking2006	OEH	Singlebeam	2006
PortHacking2001	OEH	Singlebeam	2001
GeorgesRiver1993	OEH	Singlebeam	1993
CooksRiver1989	OEH	Singlebeam	1989
SydWat	OEH	Singlebeam	Before 2015
All datasets were compiled or resampled to a grid size of 10 m.			

**Table 3:** Hawkesbury River compilation datasets.

Dataset	Data Provider	Data Acquisition Method	Date of Acquisition (if known)
5 m digital elevation model (Hawkesbury)	GA	Topographic Lidar	2011
WyongOffshore1986	OEH	Singlebeam	1986
snb_2mby	OEH	Multibeam	Before 2015
snbs_2mby	OEH	Multibeam	Before 2015
BrokenBay1978	OEH	Singlebeam	1978
CC_LADS	OEH	Bathymetric Lidar	2011
avoca_5m	OEH	Bathymetric Lidar	Before 2015
Sydney Offshore 2008	OEH	Singlebeam	2008
Broken_Bay_1977_1978	OEH	Singlebeam	1978
Hawkesbury_1987_1988	OEH	Singlebeam	1988
Hawkesbury_1983_1985	OEH	Singlebeam	Before 2015
Hawkesbury_1978_1980	OEH	Singlebeam	1980
Brooklyn_2006	OEH	Singlebeam	2006
Berowra_1995	OEH	Singlebeam	1995
HawkesburyRiver_2011	OEH	Singlebeam	2011
HawkesburyRiver_1985	OEH	Singlebeam	1985
BrisbaneWater1989	OEH	Singlebeam	1989
BrisbaneWater2004	OEH	Singlebeam	2004
BrisbaneWater1993	OEH	Singlebeam	1993
HI341_HSDB_T0001_SD_100035103	OEH	Singlebeam	Before 2015
100004058_m	OEH	Singlebeam	Before 2015
SCHOOL_11_99_HSDB_T0001_SD_1000031002_WGS84z56	OEH	Singlebeam	Before 2015
All datasets were compiled or resampled to a grid size of 50 m.			
